# Duodenal Webs: An Experience With 18 Patients

**Published:** 2012-04-01

**Authors:** Yogesh Kumar Sarin, Akshay Sharma, Shalini Sinha, Vidyanand Pramod Deshpande

**Affiliations:** Department of Paediatric Surgery, Maulana Azad Medical College, New Delhi, INDIA

**Keywords:** Duodenal web, duodenal atresia, windsock diaphragm

## Abstract

Aim: To describe the management and outcome of patients with duodenal webs, managed over a period of 12 ½ years in our unit.

Methods: It is a retrospective case series of 18 patients with congenital duodenal webs, managed in our unit, between 1999 and 2011. The medical record of these patients was retrieved and analyzed for demographic details, clinical presentation, associated anomalies, and outcome.

Results: The median age of presentation was 8 days (range 1 day to 1.5 years). Antenatal diagnosis was made in only 2 (11.1%) patients. The commonest presentation was bilious vomiting. Associated anomalies were present in 8/18 patients, common being malrotation of gut. Down’s syndrome was seen in 2 patients and congenital heart disease in 1 patient. One patient had double duodenal webs. There was a delay in presentation of more than 5 days of life in 11/18 (61%) patients. Three patients who presented beyond neonatal age group had fenestrated duodenal membranes causing partial obstruction. In addition, the diagnosis was missed in patients operated for malrotation elsewhere (n=2), imperforate anus (n=2) and esophageal atresia with tracheo-esophageal fistula (n=1). A lateral duodenotomy with excision of the obstructive membrane was done in all patients. A trans-anastomotic tube (TAT) for enteral feeding was used in 8 patients The mortality rate was 4/18 (22%); the main causes being sepsis, prematurity, very low birth weight and associated congenital anomalies. The mean hospital stay for the 14 survivors was 18 days. Total parental nutrition (TPN) was not given to any patient.

Conclusions: Congenital duodenal webs are different as the diagnosis is often missed especially in case of perforated webs. Outcome depends upon the time of presentation and associated anomalies. The use of TAT feeding for nutritional support is an easy alternative to TPN.

## INTRODUCTION

Gray and Skandalakis have classified congenital duodenal obstructions into 3 types- I, II, and III [1]. The type I or membranous duodenal obstruction (mucosal diaphragm, or web) has been quoted to account for 0.8-92% of all cases [2-5]. We analyzed 18 patients with type I duodenal obstruction, operated in our unit in the last 12½ years in order to bring forth distinctive features, the difficulties encountered in diagnosis, and final outcome. A distinct pattern of clinical behavior was noted that highlighted the need to deal with this group of patients separately from the other subtypes.

## MATERIALS AND METHODS

A retrospective study was performed by retrieving the medical records of children who underwent surgery for duodenal obstruction in one of the two units of the Department of Pediatric Surgery in a busy public tertiary-care hospital. A total of 57 children with congenital duodenal obstruction were managed over a period of 12½ years (1999 to 2011). Of these, 18 (31%) had Type I duodenal obstruction. This group was studied in detail with respect to the demographic details, antenatal diagnosis, clinical presentation, associated anomalies, delay in diagnosis, surgical procedure, and outcome of these patients.

## RESULTS

Demographic Details: The age distribution is shown in Table 1. The median age at presentation was 8 days (range 1 day to 1.5 years). There were 13 neonates. The mean age at presentation for the neonates was 4 days. Four patients were born prematurely (32-34 weeks). There was no sex predilection with the M: F ratio being one.

**Figure:**
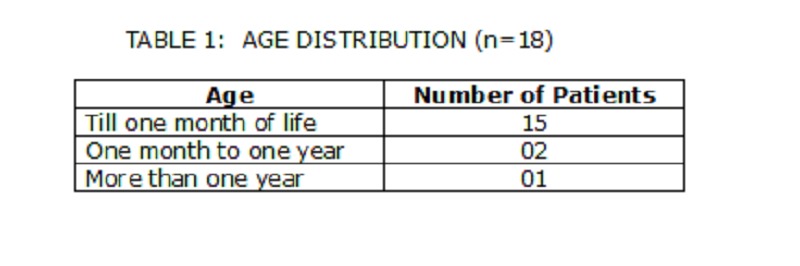
Table 1: Age distribution(n=18)

Antenatal Diagnosis: Though majority of the pregnancies were supervised, an antenatal diagnosis of duodenal obstruction on ultrasonography was made in only two patients (11%). One mother was diagnosed to have oligohydramnios. One pregnancy was entirely unsupervised with a history of intake of some traditional medicines for joint pains throughout. Of the two patients diagnosed antenatally with duodenal obstruction, only one was referred to the hospital soon after birth.

Clinical Presentation: The commonest presentation was vomiting after feeding, which was bilious in majority of the children. Two patients had vomited as long as 6 months and 1 year before medical help was sought. Two patients presented with imperforate anus. One patient with associated esophageal atresia and tracheoesophageal fistula (EA with TEF) presented with frothing from the mouth. More than 3/4ths of our children ((76.9%) had weights >3 SDs below normal at the time of presentation.

Associated Anomalies: The associated anomalies are shown in Table 2. The most common association was malrotation of gut (3/18). High anorectal malformation was seen in two patients in whom the duodenal obstruction was missed initially, but detected subsequently in the postoperative period. Ventricular septal defect was seen in only one patient. One patient was found to have two duodenal webs. Down’s syndrome was seen in only two patients.

**Figure:**
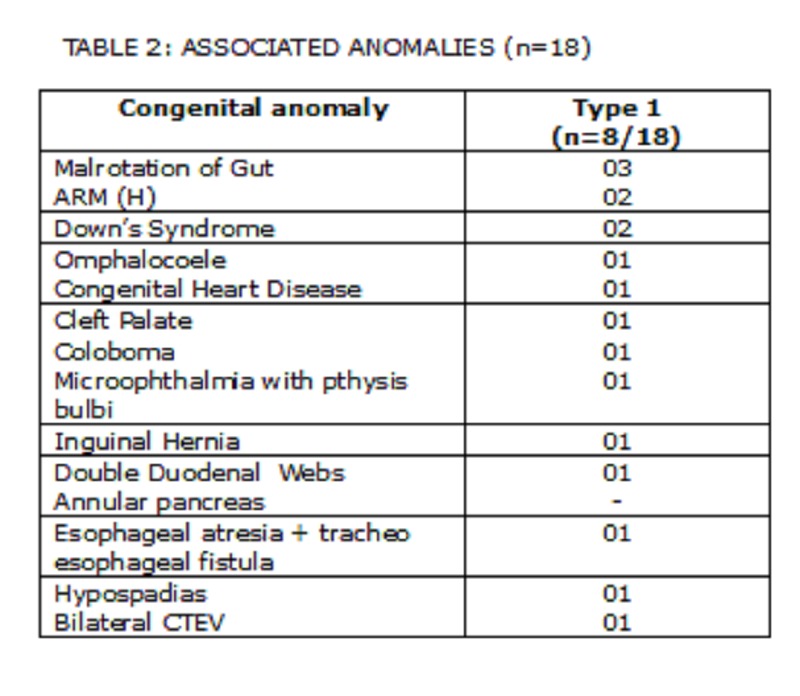
Table 2: Associated anomalies(n=18)

Delay in Diagnosis: Eleven out of 18 patients (61%) presented to us after day 5 of life. Majority were due to delay in diagnosis and ignorance of parents. One child who had double bubble appearance on antenatal ultrasound was discharged from nursery without pediatric surgical consultation and returned on day 7 of life with bilious vomiting. In 2 children with imperforate anus, the diagnosis of duodenal obstruction was made only when they had feed intolerance after diverting colostomy. The two children with associated malrotation had undergone Ladd’s procedure elsewhere and the duodenal web was missed. Three patients who presented beyond neonatal age group had fenestrated duodenal membranes causing partial obstruction.

Investigations: In majority of the cases, plain abdominal roentgeno-grams were diagnostic (double-bubble appearance) (Fig. 1); upper gastrointestinal (GI) contrast study was done in only 2 patients who presented at 7 months and 1½ years of age (Fig. 2).

**Figure:**
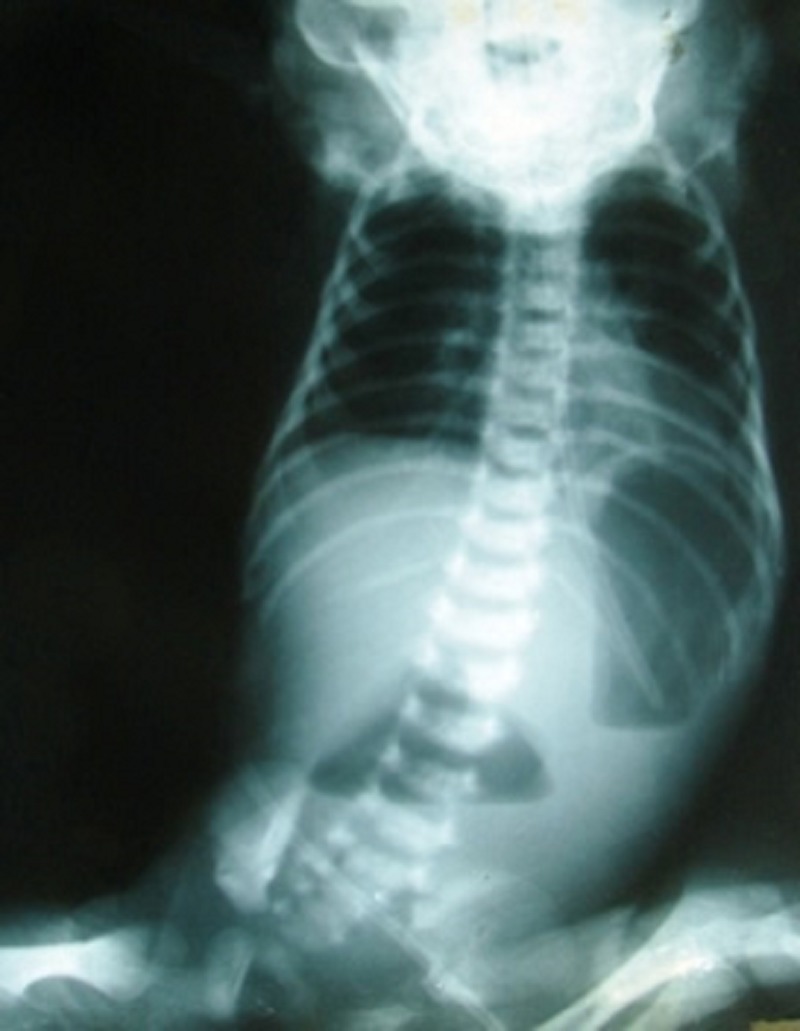
Figure 1: The double bubble sign

**Figure:**
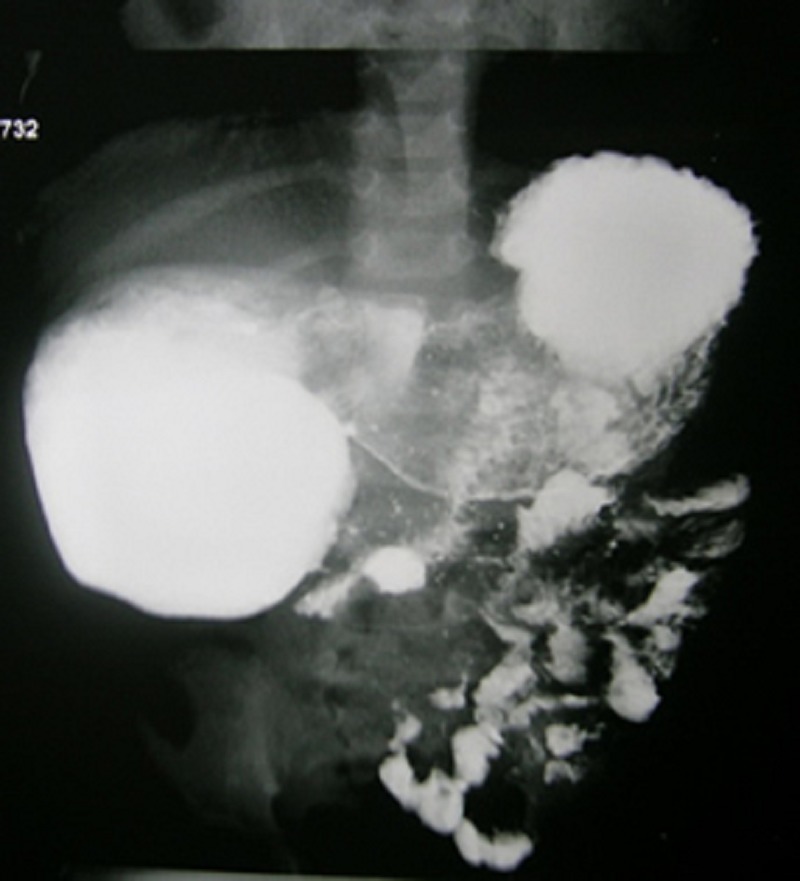
Figure 2: Upper G.I. contrast study giving suspicion of fenestrated duodenal membrane in its 2nd part.

Surgery: After an initial assessment of associated congenital anomalies and hemodynamic stabilization, the patients underwent laparotomy through a right upper quadrant transverse incision. A lateral duodenotomy with excision of the obstructive membrane was done in all patients (Fig. 3). The duodenotomy was closed transversely using 6’0 or 5’0 interrupted delayed absorbable sutures in a single layer. The location of the web was between the first and second parts of duodenum in all except two, in whom the membrane was located at the duodeno-jejunal flexure. The presence of the ‘windsock’ deformity was seen in one patient. Ladd’s procedure (without appendectomy) was done for associated malrotation in one patient; two others had the procedure done elsewhere. An imbrication procedure on the dilated proximal mega duodenum was also done in one patient. A trans-anastomotic tube (TAT) for enteral feeding was used in 8 patients. No significant intra-operative surgical or anesthetic difficulties were encountered. It was possible to visualize the exact location of the membrane in the duodenum by careful inspection during surgery. Post operative ventilatory support and supportive intensive care were required in three patients.

**Figure:**
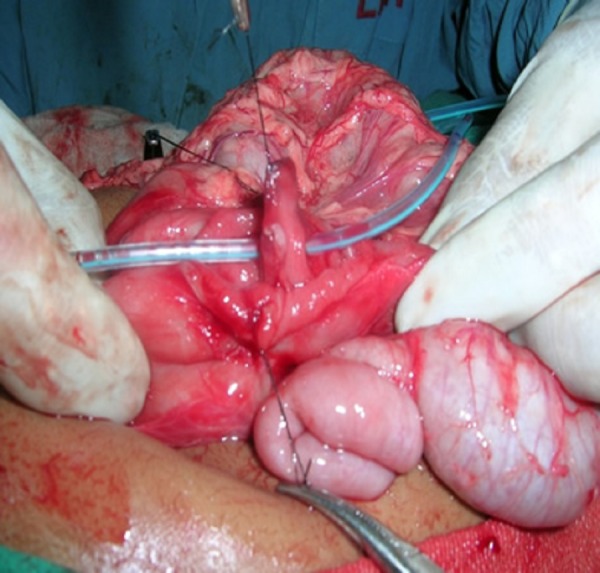
Figure 3: Intra operative picture showing the central fenestration through which an infant feeding tube has been passed.

Outcome: There were 4 deaths in the postoperative period giving a mortality rate of 22%. The causes of death were sepsis with refractory shock (n=2), aspiration (n=1), and necrotizing enterocolitis necessitating re- exploration (n=1). Three out four deaths were seen in preterm babies. Only one of the 4 preterm babies in this series survived. The mean hospital stay for the survivors was 18 days (range 11-25 days). The mean time taken to achieve oral feeds ad lib in these 14 patients was 10 days (range 7 to 13 days). Two patients survived fulminant Klebsiella sepsis. Total parenteral nutrition (TPN) was not used in any of the patients in our series. The 8 patients in whom TAT was used for nutritional support had an earlier establishment of normal oral feeding pattern. For lack of numbers, any test of statistical significance could not be performed.

## DISCUSSION

Few studies have reported duodenal membranes as a separate entity with only occasional case reports from India [5-9].

The incidence of duodenal obstruction has been reported as 1 in 6000 - 10000 live births by various authors. In a series of congenital intrinsic duodenal obstructions by Fronkalsurd et al, atresia was reported in 49%, membranes in 41% and stenosis in 10% cases [3]. Reports of different authors vary quoting the incidence of duodenal membranes from 0.8% to 92% [2,4,5]. In our series, the incidence of type I duodenal obstructions was found to be only 31% which is much less than that reported in recent literature.

In a population based study of small intestinal atresia and stenosis over a 15 year period, Forrester et al have described the incidence of congenital duodenal obstructions as 1.3/ 10,000 live births [10]. This study was done in Hawai where 75% of the population was Asian or Pacific Islanders and only 25% were Caucasians. However, they have not mentioned duodenal membranes separately in their study. No such studies have been reported from the Indian subcontinent.

Antenatal diagnosis of congenital duodenal obstruction on ultrasonography is made by the presence of double-bubble appearance and polyhydramnios. Waever et al have reported that prenatal ultrasonography picked up an abnormality in 90% of cases (n=40) with duodenal atresia [4]. In our series, only 2 patients had an antenatal diagnosis of intrinsic duodenal obstruction. Although this congenital anomaly, even if picked up antenatally does not warrant medical termination of pregnancy, early induction of labor, Caesarian section or fetal intervention, but an antenatal detection may suggest delivery in a tertiary centre where surgical correction could be done postnatally without any delay.

As seen in three of our patients, those with fenestrated duodenal membranes may present as late as late infancy or childhood or occasionally even in adulthood [12]. The fenestrated membrane may be choked with the food residue / foreign body. Another cause of delayed presentation is the gradual onset of atony and ineffective peristalsis in the dilated proximal segment of duodenum along with the development of patulous pylorus [6,12-14]. An upper GI contrast study is needed in older children to diagnose a partial duodenal obstruction as was seen in two of our patients.

Although some studies have reported non-bilious vomiting as the most common presenting feature, majority of our patients had bilious vomiting indicating that the site of obstruction was post-ampullary [6,15]. A plain X-ray abdomen with a characteristic ‘double-bubble’ sign was diagnostic in most neonates. The most common site of location is between the first and second parts (85%) [15]. In a series of 10 patients, Rowe et al have described the location of a windsock anomaly to be preampullary in 40% of cases [8].

The delay in diagnosis was mainly due to fenestrated membranes, or missed diagnosis in children with associated lower G.I. obstruction requiring surgical intervention. About half of our patients were brought beyond 1 month of age. In our series, 2 patients were operated elsewhere for malrotation during which the duodenal web was missed. There are several reports in the early 20th century where intrinsic duodenal obstruction has been missed during the Ladd’s procedure [8]. Inability to pass a stiff catheter into the duodenum should raise the suspicion of a duodenal web and demands a careful inspection. Rowe et al described 10 patients with windsock anomaly of the duodenal. Only 25% of patients with duodenal web and associated low intestinal obstruction (n=4) had classical findings on X-Ray preoperatively. All were diagnosed on upper GI contrast series done for persistent vomiting after correction of low intestinal obstructions [8]. In our series there were 2 patients with imperforate anus and 1 patient with esophageal atresia in which the diagnosis of duodenal web was missed at the time of initial surgery.

One case of double duodenal web in this series emphasizes the need for mandatory checking of distal patency of gut at the time of surgery for bowel atresia. Only 32 cases of double duodenal webs have been reported in literature till date to the best of our knowledge out of which 2 were reported in adults [16-23]. Reid in his study of 140 patients of intrinsic duodenal obstructions, found only four double duodenal intrinsic obstructions, of which two were due to webs [16]. Stinger et al have reported four patients with double duodenal obstructions of which two were due to webs [17].

The duodenotomy should be made in the lateral wall in the distal segment near the membrane. The membrane should be excised with electrocautery leaving the medial part in order to avoid injury to the Papilla of Vater. A proximal ‘mega-duodenum’ (duodenal diameter of 5 cm or more) may require imbrications or a tapering duodenoplasty procedure to avoid prolonged duodenal ileus [24].

The use of TAT for enteral feeding is controversial with no advantage being noted by some authors [4,25]. However recent reports have emerged enumerating the benefits of TAT feeding [26]. This was used in 8 of our patients all of whom had early institution of oral feeding. Prolonged duodenal ileus may sometimes persist for sometime post operatively; the use of TPN or TAT feeding may benefit this group of patients. The use of prokinetic agents is of doubtful value in these patients. Persisting signs of duodenal obstruction for more than three weeks post operatively should raise doubts of residual or incomplete excision of the duodenal membrane. This has to be confirmed radiologically before going in for a re-exploration.

With advancement in pediatric intensive care and anesthesia the survival rates for duodenal obstructions have improved to 90- 95% in the developed world [27, 28]; the major causes of mortality being associated life threatening congenital anomalies. However, there is no available data from the developing countries where a combination of life threatening congenital anomalies, pre-maturity, low birth weight and sepsis are considerable contributing factors to mortality. Crowded nurseries, nosocomial infections, cross infections and poor infra-structures add to the insult.

## CONCLUSION

We infer that although the facilities for antenatal ultrasonography are amply available even in our country, the pick-up rates for congenital abnormalities are dismal. Another indicator of poor antenatal management is the high incidence of very low birth weight babies especially in those coming from the low socio- economic strata that comprises 3/4th of our population. A large number of childbirths in India are still conducted by untrained ‘dais’ (midwives) at home with a high incidence of perinatal sepsis. This also leads to undue delay in the surgical management of such compromised neonates, thus affecting the outcomes adversely. The diagnosis may be missed by the trained pediatric surgeons even at surgery. The outcome in patients with duodenal membranes is further marred by the high incidence of associated anomalies. Further, the provision of TPN in Public Hospital nurseries is a farfetched reality. However, nutritional supplementation in these surgical neonates through TAT provides a good alternative and is strongly advocated.

## Footnotes

**Source of Support:** Nil

**Conflict of Interest:** None declared

